# Editorial: Nutrition and metabolism in kidney diseases

**DOI:** 10.3389/fnut.2023.1088977

**Published:** 2023-02-03

**Authors:** Cassiana Regina de Góes, Barbara Perez Vogt, Annabel Biruete, Thomas J. Wilkinson, Matthew Snelson

**Affiliations:** ^1^Federal University of Viçosa, Rio Paranaiba Campus, Rio Paranaiba, Brazil; ^2^Graduate Program in Health Sciences, Medicine Faculty, Federal University of Uberlandia, Uberlândia, Brazil; ^3^Department of Nutrition Science, Purdue University, West Lafayette, IN, United States; ^4^Leicester Diabetes Centre, University of Leicester, Leicester, United Kingdom; ^5^Department of Diabetes, Monash University, Melbourne, VIC, Australia

**Keywords:** acute kidney injury, body composition, chronic kidney disease, kidney replacement therapy, physical exercise, sarcopenia, protein-energy wasting (PEW)

## Introduction

The impairment of kidney function, which occurs in chronic kidney disease (CKD) and acute kidney injury (AKI), promotes specific alterations in nutrient metabolism (1) and induces a pro-inflammatory state (2). These alterations affect the nutritional status of the patients and increase morbidity and mortality risk. Among the many factors that are associated with poor outcomes in this population, protein-energy wasting, malnutrition, and sarcopenia play a significant role.

Nutritional management in individuals with impaired kidney function varies depending on the disease severity, nutritional status, cause of disease, comorbidities, medications, and treatment methods. Therefore, understanding the available methods for assessing nutritional status, establishing dietary requirements, and strategies for preventing or treating potential nutritional derangements is essential for optimal care of patients with kidney diseases. This Research Topic focuses on recent studies exploring nutrition and metabolism in CKD.

## Energy requirements and CKD

In individuals with CKD, energy requirements have traditionally been considered to be higher based on early nitrogen balance studies. However, more recent studies have questioned these higher recommendations (3–5). Indirect calorimetry is the gold standard for measuring resting energy expenditure (REE); however, its availability in clinical settings is limited. Ramos-Acevedo et al., performed a study in individuals with CKD stages 3–5 without kidney replacement therapy, incorporating measures of nutritional status and other clinical variables. They found good concordance between their models and others validated in CKD. The authors concluded that clinicians should consider using formulas that include nutritional status and other variables, such as weight, fat-free mass, comorbidities, sex, and age, to estimate energy requirements.

Similarly, Abi et al. utilized indirect calorimetry to measure REE in patients with stage 3–5 CKD and assess the relationship with the adipokines leptin, IL-6 and adiponectin in serum. In an initial analysis, the authors found that REE was positively correlated with leptin in males and females and negatively correlated with adiponectin in males only. However, when fat mass was accounted for using a multivariate linear regression model, the only significant relationship observed was between REE and leptin in males. This study highlights the importance of considering the degree of adiposity when studying adipokines, as well as the possibility of a sex-specific relationship between adipokines and energy expenditure in CKD patients.

## Plasma biomarkers in CKD

Cardiovascular mortality is increased in populations with CKD, and there is considerable interest in how commonly assessed lipid risk factors can be utilized to understand and modulate cardiovascular risk (6). In a large retrospective study of Chinese peritoneal dialysis (PD) patients, Wu et al. observed that there was a U-shaped relationship between LDL cholesterol levels and cardiovascular mortality. When a subgroup analysis was performed, this *U*-shaped relationship only remained significant in those with serum albumin of <36 g/L, used as a biomarker of malnutrition. The authors concluded that nutritional status modifies the relationship between LDL cholesterol and cardiovascular mortality in PD.

Lecamwasam et al. compared clinical and metabolomic data between patients with diabetes and early or late-stage CKD. In this study, the authors found no difference in LDL cholesterol between groups, although low-density lipoprotein triglyceride (LDL-TG) was increased in the late CKD group. The ratio between Apolipoprotein B1 and Apolipoprotein A1 (ApoB/ApoA1), a well-established risk factor for cardiovascular disease (7), was increased in the late CKD group, driven by a reduction in ApoA1. These findings suggest that apolipoproteins may be useful for assessing cardiovascular risk in high risk populations such as CKD patients.

Trimethylamine *N*-oxide (TMAO) is a uremic toxin which has been implicated in cardiovascular disease (8). Murray et al. fed pigs a highly heat-treated diet, high in resistant protein and found a profound alteration in the composition of the gut microbiota and changes in the plasma metabolome. They found that the resistant protein diet increased plasma TMAO and reduced plasma acetate, a beneficial short-chain fatty acid that is produced predominantly by the gut microbiota. This provides emerging evidence for the role of dietary resistant protein modulating the gut microbiota in the pathogenesis of cardiovascular disease in CKD.

## Adverse body composition in CKD and interventions to improve it

Sarcopenia is highly prevalent in patients living with CKD (9), yet a reliable and simple means to assess it is lacking. The product of serum creatinine and the estimated glomerular filtration rate based on cystatin C was recently proposed as a sarcopenia index. Lin et al. evaluated the sarcopenia index in 297 patients with non-dialysis stage 3b-5 CKD. They found the sarcopenia index had acceptable discriminative ability to detect sarcopenia. As such, sarcopenia index could be used as a surrogate marker for sarcopenia and may be helpful for screening in advanced CKD.

Aside from measuring body composition, bioelectrical impedance analysis also provides the phase angle, a composite marker influenced by hydration and integrity of the body cell membrane (10). Reis et al. evaluated whether the phase angle could be used as a nutritional marker and predictor of mortality in PD patients. Their findings revealed an inverse correlation between the phase angle and coronary artery calcium score, a predictor of the incidence of acute myocardial infarction and death from cardiovascular disease.

Oral nutritional supplementation (ONS) is one of the interventions recommended to manage muscle wasting in patients on maintenance hemodialysis if diet alone does not provide sufficient energy and protein intake (11). In a randomized controlled trial of 56 patients with protein energy wasting, Sahathevan et al. showed improvement in muscle mass parameters assessed by ultrasound after a 6-month ONS compared to nutritional counseling. In a small open randomized pilot trial, González-Ortiz et al. showed both intradialytic or at home ONS improved not only nutritional parameters, such as body mass index and normalized protein nitrogen appearance, as well as sleep quality in 23 patients on maintenance hemodialysis.

Obesity is also a critical issue in the CKD population. Post-transplant increases in fat mass are usually associated with insulin resistance and cardiovascular risk factors in kidney transplant recipients (12). Another intervention approached in this Research Topic was a personalized digital health intervention, with the aim to prevent weight gain after kidney transplantation. Castle et al. showed this intervention was feasible and acceptable for recent kidney transplant recipients.

## Malnutrition and diet management

Malnutrition may also be associated with kidney-related outcomes, such as increased susceptibility to AKI (13, 14). A study by Liang et al. observed that elderly patients with malnutrition, assessed by the Controlling Nutritional Status score (CONUT score), who underwent percutaneous coronary intervention, had a 2-fold increased adjusted risk of contrast-associated AKI, compared to those with no malnutrition.

Zinc is an essential micronutrient involved in numerous metabolic processes. AKI is associated with low plasma zinc (15), but outcomes with zinc supplementation in critically ill patients with AKI remain limited. Xia et al. investigated the effectiveness of zinc supplementation in 9811 patients with AKI. They found zinc supplementation was associated with improved survival in critically ill patients with AKI. Whilst further study is needed, this study highlights potential benefits of zinc supplementation in critically ill patients with AKI.

Diet management plays a crucial role in treating potential or ongoing nutritional deficiencies and derangements in patients undergoing hemodialysis (11). However, current dietary recommendations may not be culturally appropriate. In a qualitative study, Song et al. conducted semi-structured interviews with 23 patients in China on hemodialysis to explore their perceptions and attitudes toward diet. Findings showed diet behavior in Chinese patients undergoing hemodialysis is strongly influenced by culture. Culturally sensitive interventions regarding the improvement of diet intake are urgently needed.

Healthy lifestyle behaviors, including a healthy diet, weight control and physical activity promote the kidney health of children, reducing long-term kidney damage in adulthood (16). Physical activity may have an impact on hydration status and kidney health, but the interaction of hydration status and physical activity level on kidney function is not well-studied in children. Li et al. explored associations of kidney damage with the interaction of hydration status and physical activity level in 1,914 primary school children from China. They found longitudinal interactions of hydration status and physical activity level on early kidney damage and found increased dehydration among the children over time. These results support the importance of adequate water intake, and suggest that children can be protected from early kidney damage by euhydration, either with sufficient or insufficient physical activity.

## Perspectives

In summary, the studies included in the Research Topic on “*Nutrition and metabolism in kidney diseases*” enrich our understanding on a wide variety of topics, from nutritional assessment aspects, such as energy expenditure, body composition, bioelectrical impedance analysis parameters, and new and traditional biomarkers evaluation, to nutritional management of the main nutritional disorders in CKD and AKI, including malnutrition, sarcopenia, macro And micronutrients deficiency, and obesity. The articles included in this collection highlight future research directions, assist in the development of novel therapeutic approaches, and contribute to improvements in clinical practice.

## Author contributions

All authors listed have made a substantial, direct, and intellectual contribution to the work and approved it for publication.
